# Structures and practices of whistleblowing in healthcare: An integrative review

**DOI:** 10.1177/09697330261449295

**Published:** 2026-05-20

**Authors:** Outi Järvinen, Riitta Suhonen, Johanna Wiisak

**Affiliations:** 1 Department of Nursing Science, 8058 University of Turku, Turku, Finland; 2 Turku University Hospital, The Wellbeing Services County of Southwest Finland, Turku, Finland; 3 Lero the Research Ireland Centre for Software and J.E. Cairnes School of Business & Economics, University of Galway, Galway, Ireland

**Keywords:** healthcare, integrative literature review, organisation, structures and practices of whistleblowing, wrongdoing

## Abstract

Wrongdoings occur in healthcare, and effective whistleblowing structures and practices are essential for ethically addressing these issues in organisations. However, research on whistleblowing in healthcare from an organisational perspective remains limited. The aim of this integrative literature review was to synthesise the empirical evidence about the structures and practices of whistleblowing in healthcare organisations. Whittemore and Knafl’s methodology was followed, and the study selection process was documented using the Preferred Reporting Items for Systematic Reviews and Meta-analyses flow diagram. A systematic literature search was performed in February 2024 and updated in January 2026 across Cumulative Index to Nursing and Allied Health Literature, MEDLINE (PubMed), Scopus, SocINDEX, and Web of Science Core Collection. This search was complemented by a manual search. Peer-reviewed empirical studies published in English were selected. Data were analysed using inductive content analysis. Fourteen included studies were published between 2007 and 2023. Most studies were qualitative and conducted in the United Kingdom; the overall methodological quality was high. Five categories describing the structures and practices of whistleblowing in healthcare organisations were identified: (1) inconsistent, inadequate and insufficient structures and practices of whistleblowing, (2) the absence of standardised practices or their implementation in an inconsistent manner, (3) the influence of organisational culture and leadership on whistleblowing structures and practices, (4) the need for substantial development in whistleblowing structures and practices, and (5) the need to reframe attitudes towards whistleblowing as both an opportunity and a tool for organisational improvement. The results show the fragmented and inconsistent nature of structures and practices of whistleblowing in healthcare organisations; they also underscore the influence of organisational culture, leadership, and power dynamics on their effectiveness. The findings illuminate how internal whistleblowing develops and highlight the need for future studies that employ designs capable of more robustly evaluating whistleblowing mechanisms.

## Introduction

Wrongdoings – including intentionally committed illegal or unethical practices, professionally inappropriate behaviours or omissions – occur globally in healthcare and whistleblowing is an action that aims to terminate them.^1–4^ These wrongdoings may involve violations of an individual’s humanity, dignity, life and health, with some of them meeting the criteria for criminal offences.^4–6^ In many healthcare systems, formal mechanisms for reporting concerns form part of clinical governance and primarily support routine concern raising but may be insufficient for addressing intentional wrongdoing that requires whistleblowing protections. Moreover, existing evidence suggests that where whistleblowing structures are in place, their effectiveness in ending wrongdoing and protecting whistleblowers varies considerably.^5–7^

For example, wrongdoings in the United Kingdom’s (UK) National Health Service led to a national inquiry, which highlighted the seriousness of these actions and their risks to patient safety. This prompted a reassessment of the structures and practices of whistleblowing. The resulting report initiated a widespread public debate and drew international attention to the structural and procedural challenges of healthcare systems.^1^ Despite whistleblowing legislation^8^ and healthcare professionals’ ethical codes,^9^ concerns have been raised in the literature regarding how the structures and practices of whistleblowing are implemented in healthcare settings.^10^ Hence, this organisational perspective was the focus of this review.

Effective and appropriate structures and practices to manage whistleblowing are central to ensuring the safety and well-being of both patients and healthcare professionals.^11^ When healthcare organisations establish robust structures and practices of whistleblowing, they foster a culture of accountability, trust, and ethical leadership, which supports improvements in care quality and patient safety.^11–13^ Evidence further suggests that failure to address wrongdoing and silence around concerns may contribute to staff attrition, organisational dysfunction, preventable harm, and avoidable patient deaths.^1,7,12^ Currently, healthcare organisations may tolerate arrangements that are harmful to their own interests due to shortcomings in governance or leadership practices,^14^ and may become desensitised to the pervasive presence of such harmful practices^3,15^.

## Background

In this review, ‘wrongdoing’ refers to intentionally committed illegal, or unethical acts, professionally inappropriate behaviour, or omissions in healthcare settings.^4–6,16^ In whistleblowing legislation, wrongdoing more broadly encompasses breaches of law, threats to the public interest, risks to patient safety, abuse of authority, and failures to comply with professional or regulatory standards.^8^ Whistleblowing can be understood as a moral act or a series of acts – a process aimed at ending these wrongdoings. As one act, whistleblowing entails the presence of an individual who acts independently to address illegal, unethical, or unprofessional conduct.^5,16^ As a process, whistleblowing is typically a complex, multi-stage procedure, which is initiated by either a suspicion of wrongdoing or a clear observation of it.^4,5,16^ Wrongdoing suspicions or observations are followed by extensive reasoning and deliberation.^17^ In practice, whistleblowing can be undertaken inside the organisation through designated formal whistleblowing channels or may escalate to external whistleblowing addressed to supervisory authorities or, in some cases, the media, where ordinary governance mechanisms are perceived to have failed.^4,5^ While the duty to intervene is theoretically clear for most professionals, whistleblowing requires ethical and professional competence, as well as personal abilities such as moral courage and decision-making ability.^11,14,17–19^

In this review, whistleblowing is conceptually distinguished from speaking up and routine internal reporting practices embedded in everyday clinical governance, such as incident reporting, complaints procedures, and standard line management escalation^4–6^. Whistleblowing is legally regulated^8^ and ethically driven.^9,17,19^ It can be understood as occupying a specific position along a continuum of concern raising, ranging from ordinary governance-based reporting mechanisms to external whistleblowing^4–6^. On this continuum, whistleblowing is characterised by situations in which professionals perceive that concerns cannot be safely or effectively addressed through ordinary organisational channels,^1,4,12,13^ involve heightened personal or professional risk,^17–19^ and/or require escalation beyond usual managerial pathways, including designated independent internal roles or external authorities. A healthcare professional becomes a whistleblower when they disclose suspected or observed wrongdoing in situations where ordinary organisational reporting mechanisms are perceived as ineffective or unsafe.^1,4,5,8^

Healthcare organisations need clear structures and operational practices to address emerging wrongdoing and protect whistleblowers from harm^11–13,19^. Specifically, there is a need to examine the impact of organisational culture on whistleblowing, as well as the reactions and follow-up actions of the organisational representatives managing wrongdoing. The need for formal whistleblowing practices, programmes, and interventions in healthcare has been identified^4,5,11,19^ and these are also regulated by law in many countries (see, for example^20,21^).

The present review is grounded in the challenges healthcare organisations encounter in addressing wrongdoing ethically. These challenges often prevent suspicions or observations from leading to whistleblowing and reported wrongdoing from driving meaningful changes in practice.^4^ Consequently, serious risks to patient safety, professional integrity, and organisational learning may persist. In the absence of clear and trusted organisational structures and practices for whistleblowing, individual professionals may be left to navigate complex ethical decisions on their own, relying on personal judgement rather than systematically supported processes.^7,17,19^ Several scholars have highlighted the need to examine these issues and develop appropriate structures and practices of whistleblowing from the organisational perspective.^4,6,12,17^ However, the literature presents conflicting and sometimes critical views on how reporting systems and whistleblowing practices function within healthcare organisations,^12,13^ underscoring the need for a systematic synthesis of existing empirical evidence.

## Aim

The aim of this review was to synthesise empirical evidence about structures and practices of whistleblowing in healthcare organisations. The provided knowledge can be used to advance internal whistleblowing and develop whistleblowing channels and processes in these organisations. The following question was addressed: ‘What structures and practices of whistleblowing exist in healthcare organisations?’

## Methods

### Design

An integrative review was conducted following the five steps of the methodological framework developed by Whittemore and Knafl.^22^ (1) problem-identification, (2) research strategy and literature search, (3) methodological quality assessment, (4) data analysis, and (5) presentation and synthesis of the findings. This approach enabled the systematic synthesis of heterogeneous empirical evidence across different study designs, allowing both qualitative and quantitative findings to be examined together, and was therefore appropriate for the review aim of synthesising organisational structures and practices related to whistleblowing in healthcare. The study selection process was documented using the Preferred Reporting Items for Systematic Reviews and Meta-analyses (PRISMA) flow diagram to illustrate the stages of identification, screening, and inclusion.^23^

### Search strategy

A literature search without limiters was carried out in February 2024 and updated in January 2026 on five scientific databases: the Cumulative Index of Nursing and Allied Health Literature, MEDLINE (PubMed), Scopus, SocINDEX and Web of Science Core Collection. No starting date was applied in order to capture all available empirical evidence on structures and practices of whistleblowing in healthcare. The search terms and strings were developed in collaboration with a health and medical science library informatics expert (Supplementary file 1). The following terms and keywords were applied with the Boolean operators AND or OR: whistleblowing AND (‘organisational consequences’ OR ‘organisational structure’ OR ‘organisational policy’) AND healthcare. Search builder features and MeSH terms were used when appropriate. After the records were identified, they were screened for duplicates, which were removed by the first author. Afterwards, two researchers continued to independently screen the records based on their titles and abstracts. Next, the full texts were retrieved and screened against the eligibility criteria, and studies were included when two authors reached a consensus. Finally, the reference lists of the included articles were screened manually, which produced some new articles for the review. The references were managed with the software Zotero.

### Eligibility criteria

Articles were included in the review if they (1) were peer-reviewed empirical studies that; (2) addressed misconduct or wrongdoing committed by healthcare professionals; (3) examined whistleblowing structures, practices, or reporting protocols, channels or processes; (4) in healthcare organisations; and (5) were published in English. Articles were excluded if they examined (1) misconduct by clients, such as insurance fraud, and (2) focused exclusively on individual-level perspectives, including personal opinions, experiences or the consequences of whistleblowing without addressing organisational perspective were (3) study protocols, theses, literature reviews, opinion pieces, and expert articles.

### Quality appraisal

Quality appraisal was carried out using the Mixed Methods Appraisal Tool (MMAT)^24^, which was employed on 10 qualitative, two quantitative and two mixed methods study designs. The MMAT includes two screening questions, which are followed by five items. The ranking of the items comprises the options ‘yes’, ‘no’, and ‘can’t tell’. According to the screening questions, all the articles were eligible for quality appraisal. Most of the articles (n = 11) received full scores (5/5), indicating high quality. Two articles received moderate scores (3/5), and one article was rated as having low quality (2/5). No article was excluded on the basis of the quality appraisal scores; however, findings from moderate- and low-quality studies were interpreted cautiously and, where possible, corroborated by high-quality studies.

### Data synthesis

The data from the included articles were extracted and inserted into a template constructed for this review by the research team. The extracted data included the author(s), year of publication, country, aim(s), study design and sample. The data were analysed with inductive content analysis^25^ primarily by the first author, with interpretations discussed among the research group until a consensus was reached.

In the preparation phase, the articles were repeatedly read to achieve familiarity with their data, and their key characteristics and main findings were compiled into a matrix. During the organising phase, relevant content pertaining to the review questions was openly coded from the original content of the included articles. Codes with similar meanings were grouped into subcategories. In the abstraction phase, the subcategories were compared and further interpreted to identify broader conceptual patterns, which were synthesised into five higher-level categories. Given the heterogeneity of the included studies, data synthesis focused on manifest content related to organisational structures and practices of whistleblowing rather than latent interpretation. This approach enabled systematic integration of empirical findings while maintaining analytical coherence and methodological transparency across diverse study designs. MS Excel was used to support data management and the data analysis process (Table 1). Finally, the structures and practices of whistleblowing in healthcare organisations were summarised and presented in a visual format. When reporting the findings, the terminology used in the original studies (e.g. ‘raising concerns’, ‘reporting wrongdoing’ or ‘internal whistleblowing’) is retained where appropriate. Such terms reflect source level language and are interpreted analytically in line with the conceptual distinction of whistleblowing adopted in this review, rather than indicating distinct conceptual categories.

**Table 1.** An example of the data analysis process.

Original content	Code	Sub-category	Category
‘Although the organization invited staff and faculty to speak about concerns, it appeared to lack an authentic capacity for listening or a full commitment to address concerns’. (Dixon-Woods et al., 2019, p. 581)	A lack of capacity for listening	Inadequate organisational responsiveness	Inconsistent, inadequate and insufficient structures and practices of whistleblowing
“It was found that whistleblowing was only conceptually associated with service quality and patient safety, indeed, beta was not able to effectively handle its whistleblowing initiative to improve service quality’. (Ciasullo et al., 2017 p. 175)	Ineffective handling of whistleblowing initiatives		
“Nurses characterized the organizational system they practiced in as being unresponsive to their concerns about patient care quality and safety. (Attree, 2007, p. 400)	Organisational unresponsiveness to concerns		
‘The organization… lack… a full commitment to address concerns’. (Dixon-Woods et al., 2019 p. 581)	Lack of commitment to address concerns	Insufficient organisational commitment	

## Findings

### Studies retrieved

**Figure 1.**
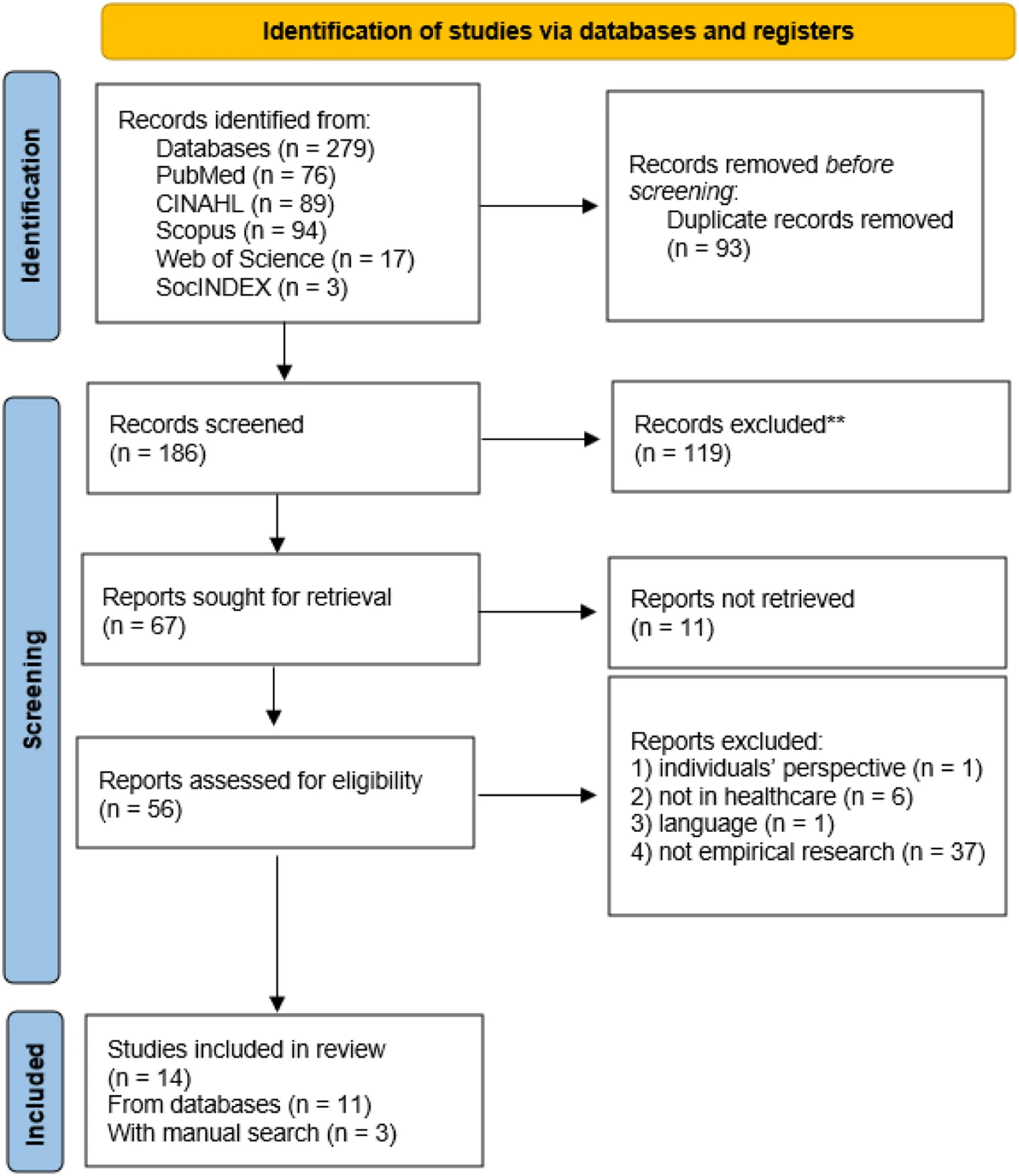
PRISMA flow diagram (page et al., 2021).

First, a literature search yielded 279 records; 93 were duplicates, and they were removed. Then, the titles and abstracts of the remaining 186 records were screened. Next, 56 full-text reports were screened for eligibility. Finally, 14 articles were included in the review (Figure 1).

### Characteristics of the studies

The studies (*n* = 14) were published between 2007 and 2023. Most (*n* = 8) of the studies were conducted in the UK,^26–33^ two were carried out in Italy,^34,35^ and one was conducted in each country, Australia,^7^ Ireland,^36^ and South Africa.^37^ One study was carried out in two countries, which were not specified^38^ (see Table 2).

**Table 2.** Characteristics of the included studies (*n* = 14) about whistleblowing structures and practices and quality appraisal scores (QAS) according to Mixed Method Appraisal Tool (MMAT, Hong et al. 2018).

Author(s), year	Country	Aim of study	Study design/methods	Setting/sample	QAS
**Qualitative studies**					Qualitative
Attree, 2007	UK	To explore factors influencing nurses’ decisions to raise concerns about care quality	Qualitative study with semi-structured interviews Grounded theory	Acute National Health Service trusts Nurses (*n* = 142)	5/5
Campra M et al., 2023	Italy	To examine the evolution of the anti-corruption system and the stakeholders’ perceptions in healthcare organisations as a new form of social responsibility	Longitudinal case study with observation, semi-structured interviews and documentation Systematic combining technique	Health organizations Observation: Health organisations (*n* = 13) Interviews: Managers (*n* = 6) Corporate anti-corruption plans: Health organisations (*n* = 13)	2/5
Ciasullo et al., 2017	Italy	To contribute to the advancement of the scientific knowledge on whistleblowing by investigating institutional, organisational, and cultural barriers to whistleblowing implementation	Qualitative study based on three case studies with unstructured and structured interviews and documentation Constant comparison	Italian national health service/healthcare organizations Unstructured interviews: Whistleblowing key informants (*n* = 15) Structured interviews: Public officers responsible for the implementation of whistleblowing (*n* = 3) Strategic and business plans, policy reports, information pamphlets, and census data: Healthcare organizations (*n* = 3)	5/5
Delpino et al. 2023	UK	To explore perceptions of a ‘Freedom to speak up guardians’ and ‘confidential contacts’ through shared experiences and personal stories	Qualitative study with focus group interview Thematic analysis with inductive approach	National health service Freedom to speak up guardians and confidential contacts (*n* = 8)	5/5
Dixon-Woods et al., 2019	UK	To understand barriers to voice and make improvements in identifying and responding to transgressive or disruptive behaviours	Qualitative case study with interviews and intervention implementation Constant comparison	Academic medicine entity Interviews: Senior leaders (*n* = 20) Frontline personnel (*n* = 47) Evaluation: Reports of disruptive behaviour (*n* = 382) In-depth reviews (*n* = 55) involving interviews (*n* = over 400) Reviews of individuals in superior positions to those affected by the behaviour (*n* = 20)	3/5
Jackson et al., 2010	Australia	To explore the reasons behind the decision to blow the whistle and provide insights into nurses’ experiences of being whistleblowers	Qualitative narrative inquiry with in-depth semi-structured interviews Categorical content analysis	Healthcare Nurse whistleblowers (*n* = 11)	5/5
Jones & Kelly, 2014	UK	To explore perceptions of whistleblowing in older people’s care	Qualitative narrative inquiry with individual and focus group semi-structured interviews Thematic analysis	Healthcare and social care/older peoples’ care Individual interviews: Registered nurses (*n* = 9) Nurse managers (*n* = 5) Care assistants (*n* = 23) Nurse students (*n* = 16) Regulators/police (*n* = 4) Focus group 1: Registered nurses (*n* = 3) Care assistants (*n* = 3) Focus group 2: Nurse students (n = 8) Focus group 3: Nurse students (n = 8)	5/5
Martin et al., 2018	Two countries, not specified	To examine the role of formal channels in encouraging or inhibiting employee voice about concerns	Qualitative study with interviews Constant comparison	Two countries, three academic hospitals Leaders and senior managers (*n* = 57) Frontline personnel (*n* = 108)	5/5
Martin et al., 2019	UK	To examine the experiences of clinical and managerial leaders in the English healthcare system charged with implementing policy goals of openness, particularly in relation to improving employee voice	Qualitative study with semi-structured interviews Constant comparison	National health service, regulatory and third sector organisations Key stakeholders (e.g. leaders, Freedom to Speak Guardians, and policymakers) (*n* = 51)	5/5
Martin et al. 2021	UK	To examine the role of the ‘Freedom to speak up guardian’ and its realization in practice.	Qualitative study with semi-structured interviews Constant comparison	National health service, regulatory and third sector organisations Key stakeholders (e.g. leaders, Freedom to Speak Guardians, and policymakers) (*n* = 51)	5/5
**Cross-sectional studies**					Cross-sectional
Fleming et al., 2018	UK	To analyse the current knowledge and experience of surgical trainees regarding whistleblowing/reporting of concerns and available reporting systems	Cross-sectional study with questionnaire Statistical analysis	International Surgical Conference Surgical trainee delegates (*n* = 479)	5/5
Moore & McAuliffe, 2010	Ireland	To explore experiences of those who have observed poor care and have reported on it	An exploratory quantitative research design with questionnaire Statistical analysis	Hospitals across health service executive regions Nurses (*n* = 152)	5/5
**Mixed method studies**					Mixed method
Jones et al., 2022	UK	To better understand the guardian role and their implementation into national health service acute trusts and mental health trusts and whether or not they are helping workers to speak up about their concerns	Mixed methods study with semi-structured interviews and six case studies including in-depth interviews, observations and documentation Thematic analysis and an inductive data condensation process	Acute hospital trusts and mental health trusts Semi-structured interviews: Guardians (*n* = 87) In-depth interviews: Key stakeholders (mostly senior leaders but also trade union representatives and workers who had spoken up to the guardian (*n* = 109) Observations 240 h: Non-participant observations of meetings and interactions involving the guardians Documents (e.g. board reports, minutes, agendas, and newsletters)	3/5
Kusu-Orkar et al., 2018	South Africa	Threefold aim to (i) elicit the views of medical students and doctors regarding whistleblowing, (ii) add to the current discussion and evidence base on whistleblowing in the South African healthcare system, and (iii) contribute to the current research base on whistleblowing in developing countries	Mixed method study with questionnaire including free text and closed questions Thematic analysis and descriptive statistics	A public hospital Surgeons (*n* = 40) Medical students (*n* = 14) Anaesthetists (*n* = 3) Internal medicine doctor (*n* = 1)	5/5

The study design was qualitative in 10 studies,^7,26–28,30,32–35,38^ of which three were case studies.^28,34,35^ A cross-sectional design was used in two studies,^29,36^ and a mixed methods design was employed in two studies.^31,37^ Interviews were used for data collection in seven studies,^7,26,27,30,32,33,38^ while two studies used questionnaires.^29,36^ Multiple methods were used in five studies,^28,31,34,35,37^ including both interviewing and documentation in four studies,^28,31,34,35^ and observation in two studies.^31,34^ In one mixed methods study, the data were collected with questionnaires, which included both open and statistical data.^37^ The data were analysed with constant comparison in five studies,^28,32,33,35,38^ thematic analysis in four studies^27,30,31,37^ and statistical analysis in three studies.^29,36,37^ In one study, grounded theory was used^26^ and in another, categorical content analysis was employed^7^ (see Table 2).

Apart from one study, for which the data were collected at a conference,^29^ all the studies were conducted in healthcare settings at the national level. One study was carried out in academic hospitals in two countries,^38^ and two studies were conducted in multiple settings, including healthcare, regulatory, and third sector organisations.^32,33^ Nearly all the studies involved a mix of professionals, apart from four, of which three included only nurses^7,26,36^ and one included surgical trainee delegates.^29^ Managers were involved in seven studies,^30,32–36,40^ speak up guardians in four studies,^29,32–35^ and policy makers in two studies,^32,33^ among others. In the qualitative studies, the number of participants ranged between 6 and 165; in the quantitative studies, it ranged from 152 to 479, and in the mixed methods studies, it ranged between 58 and 198. The samples of some studies also included documents with their numbers ranging from 13 to 382 (Table 2).

### The structures and practices of whistleblowing in healthcare organisations

Five categories describing the structures and practices of whistleblowing in healthcare organisations were identified: (1) inconsistent, inadequate and insufficient structures and practices of whistleblowing, (2) the absence of standardised practices or their implementation in an inconsistent manner, (3) the influence of organisational culture and leadership on whistleblowing structures and practices, (4) the need for substantial development in whistleblowing structures and practices, and (5) the need to reframe attitudes towards whistleblowing as both an opportunity and a tool for organisational improvement.

#### Inconsistent, inadequate, and insufficient structures and practices of whistleblowing

Structures and practices of whistleblowing were frequently inconsistent, inadequate, and insufficient, and this was often reflected as inappropriate organisational attitudes towards whistleblowing and whistleblowers.^7,26–29,36^ Attitudes could be directly hostile,^28^ varying,^27^ indifferent,^25,29^ or inadequate.^7,36^ It was common for raised concerns to be disregarded or left unaddressed.^26,28,36^ Negative responses to whistleblowing were found in organisations characterised by a closed climate and where the concept of whistleblowing was viewed unfavourably.^26,30^

Healthcare personnel frequently perceived formal whistleblowing channels as cumbersome, complex, and unreliable. The lack of clarity regarding accountability in these channels posed significant challenges for professionals seeking a secure means to raise concerns.^27,28^ For complex concerns, problems arose when they had to be first converted into legally verifiable documents for formal channels.^38^ Significant issues were identified in existing whistleblowing channels, highlighting the need for improvements to enhance patient safety. Formal procedures may stifle whistleblowing, especially regarding less concrete worries; this render disclosures ‘dangerous’, thus contradicting the intended purpose of the systems in question.^29,38^ For example, many medical trainees reported feeling unable to raise concerns due to perceived barriers and lack of confidentiality.^29^

Power dynamics and hierarchies in healthcare organisations influence whistleblowing practices, which result in the unequal application of agreed protocols across organisational levels. For instance, challenges in raising concerns were especially noted regarding ‘untouchable’ individuals in high revenue-generating positions.^28,36,37^ These individuals were often medical members of staff and could engage in norm-violating behaviour with impunity.^28^ Other significant interpersonal relationships shaped perceptions of who was considered reportable.^36,37^ Individuals in lower positions were often fearful of challenging powerful figures and their allies. Organisations lacked well-coordinated processes to identify, evaluate, and address disruptive behaviour among physicians.^28^ Physicians also reported fear of supervisors, which harmed patient care and hindered positive organisational change.^37^

### The absence of standardised practices or their implementation in a highly inconsistent manner

Standardised whistleblowing practices were often absent or inconsistently applied, while whistleblowing channels and reporting procedures were widely perceived as ambiguous.^26,28,29,37^ For example, these procedures were at times obscure or unknown, which left individuals uncertain and poorly informed about available systems and guidelines.^26,29^ In some cases, formal reporting systems were absent, though employees presumed their existence.^37^ Systems were sometimes described as closed and secretive, with individuals believing that they were discouraged from reporting concerns, problems and errors.^26^ Whistleblowing channels were often unknown in organisations and inadequately communicated.^29^ In the absence of established systems and practices, the need for a formal reporting structure was emphasised.^37^

Fear of lawsuits, complaints, and union involvement led leaders to formalise whistleblowing for legal compliance, but this sometimes hindered the identification of critical issues, as formal reporting risked damaging professional relationships.^38^ Informal channels were present in some cases,^7,36,38^ as well as anonymous reporting options, which increased the willingness to disclose misconduct.^35^ Informal reporting depends on leader accessibility and confidential supervisor-employee relationships, with some organisations supplementing formal systems by encouraging open reporting without formal procedures.^38^ Informal channels were deemed essential for complex cases where whistleblowers preferred non-documented reporting.^36^ Occasionally, informal collective intervention by a team was perceived as a safe option to address issues, especially those related to patient safety and organisational errors.^7,38^ Often whistleblowing channels organically developed into a complex, poorly coordinated and organisation-specific ‘ecosystem’.^33^

Gaps were observed between established protocols for whistleblowing and actual practices, resulting in a failure of processes to function as intended.^28,35^ For example, an organisation may encourage staff to raise concerns while simultaneously lacking the capacity to genuinely listen or commit to addressing the issues raised.^28^ Although whistleblowing was conceptually tied to service quality and patient safety in organisational goals, it was not practically utilised to enhance service quality.^35^

#### The influence of organisational culture and leadership on whistleblowing structures and practices

The dynamics of whistleblowing were connected to the prevailing organisational culture, leadership practices, and overall management approaches, which can be considered central factors in shaping whistleblowing practices.^7,26-30,32,35^ Efforts to elevate care quality and safety are essential, but they remain mere rhetoric without organisational cultural change.^26,35^ Organisations were urged to cultivate openness and communication at all levels through a transparent culture and clear reporting mechanisms, which enable staff, including trainees, to raise concerns at an early stage and engage in dialogue that may reduce the need for whistleblowing.^29,30^.A culture of open learning,^26,27^ as well as a generally open, ethical, and confidential atmosphere,^26,30,32,35^ were identified factors supporting effective whistleblowing. Trust in managers and the reporting system facilitated open discussion.^26^ Furthermore, successful practical improvements encouraged a reflective culture of learning from mistakes.^27^

In organisations where challenges in whistleblowing practices were encountered, negative organisational cultures, such as closed, concealing, accusatory, inward-looking, isolating, obstructive, silence-focused or fear-laden cultures, were reported.^7,26,29,30,32^ A negative organisational culture can lead to a distorted view of the situation, where a lack of openness^32^ and gradual emotional detachment from issues over time,^30,32^ as well as the ‘unwritten rules and norms’ of the workplace,^26^ can sometimes hinder whistleblowing. Barriers to speaking up were linked to shared experiences of the organisational culture, which may impede whistleblowing.^7,26–28,32,35^ For instance, prior adverse experiences can foster a culture of silence and closure.^32^ Likewise, in certain organisations, intense pressure to maintain silence and uphold the status quo resulted in suppression.^7^ In some cases, long-serving employees reinforced workplace norms that inhibited the raising of concerns.^30^ In other cases, the unit’s professional culture appeared to discourage the reporting of wrongdoing, despite the organisation’s broader ethical climate,^35^ According to some studies, the organisational system and culture rendered raising concerns difficult, with a prevailing culture of fear being particularly detrimental to the whistleblowing process.^7,26,28^ In organisations that enforced silence and ignored issues, declining trust and fear of disrupting the status quo, as well as distrust in reporting systems, created significant barriers to whistleblowing.^26,28^

Management and leadership were regarded as central to the effectiveness of whistleblowing practices.^26–28,30,32,35,38^ Management practices were occasionally found to pose challenges to safe whistleblowing in healthcare.^27^ Unresponsive leadership was seen as stifling dialogue, which caused silence, aggression, and indifference. Managers acknowledged that limited information on their part hindered the raising of concerns in organisations.^32^ Senior leadership’s engagement with whistleblowing was seen to directly influence its implementation,^35^and leadership was generally perceived as important for a culture of openness.^30^ For example, senior leaders emphasised the importance of addressing concerns related to patient safety and care quality.^28^ Managers aimed to cultivate a communicative culture that encouraged issue reporting and underscored the value of open dialogue among staff.^30^ From a whistleblowing perspective, positive leadership involved supervisors addressing concerns respectfully, confidentially, and thoroughly, with timely and accountable investigations.^26^ Positive leadership was associated with a strong and visible supervisor, who helped raising issues.^27^ Whistleblowing effectiveness depended on supervisor leadership, which fostered safety and organisational commitment, and it required sensitivity to be integrated into quality improvement.^35^ Competent middle managers who built personal ties with frontline staff were key to hearing concerns.^38^

#### The need for substantial development in whistleblowing structures and practices

The need to substantially develop whistleblowing structures and practices was identified due to the existing ones being incomplete and evolving, among other things.^26,30,35,36^ A consistent national structure and practice for whistleblowing have been urged to support the monitoring of poor care and its trends, with a systematic approach to corrective actions promoting learning from such trends.^26,36^ Relevant organisational changes rely on the collective understanding of how whistleblowing is perceived in healthcare, which highlights the need for broad public debate.^30^

The structuring of whistleblowing in organisations was found to require clear action plans^32^ and its establishment as a widely accepted and normalised practice.^36^ Achieving these goals calls for robust and effective organisational structures that not only monitor activities but also support individuals engaging in whistleblowing.^26^ For instance, the authors of a case study showed that two organisations integrated whistleblowing into their strategies and implemented it through specific regulations.^35^ In the development and implementation of new practices, it was seen that projects could facilitate the process of organisational change.^34^

Whistleblowing practice improvement proposals often emphasise leadership to foster an ethical climate and highlight its positive organisational impacts.^35^ Supportive leadership, for example, is essential for empowering those who raise concerns about patient safety.^29,36^ Senior management should strengthen their commitment to addressing misconduct by nurturing open dialogue, supporting a learning-oriented speak-up culture, and backing whistleblowing initiatives across all levels of the organisation.^27^ Management should integrate concern-raising into organisational strategy to promote an open, supportive culture and deliberately counter organisational deafness to wrongdoing^30^; it should also embed it into organisational ethical codes to facilitate its practical implementation.^35^ Proactive, reflective meetings at all levels of management should encourage a culture of raising concerns and make this action a routine practice.^27^ Additionally, team meetings and staff training could serve to establish a more democratic and interactive culture.^30,36^ Greater visibility of supervisors in care units was also emphasised as a crucial factor.^36^

The need for training emerged as a critical factor regarding whistleblowing practices in healthcare units. Several of the authors of the selected articles emphasised that staff should be trained in unit-specific whistleblowing procedures and instructed through formal protocols on how to raise concerns.^29,35-37^ Training and induction on whistleblowing practices facilitate the development of an open culture where employees feel empowered to raise concerns. Induction also prevents the transmission of questionable practices to new employees.^30^ Furthermore, employee training is crucial for creating a supportive environment for the practical implementation of whistleblowing procedures.^35^

In a number of articles, the need to improve reporting systems was recognised.^26,36,37^ For example, the development of a national reporting system was highlighted,^26^ and it was suggested that each hospital should have a designated officer responsible for handling complaints and concerns.^36^ This officer would provide appropriate means to simplify the whistleblowing process by effectively coordinating the process when necessary.^37^ On occasion, improving whistleblowing practices was seen to require more staff.^36^ A functional reporting system was expected to be easily accessible and facilitate the expression of concerns through appropriate channels.^29^ Some authors recommended removing formal process drawbacks and creating positive environments where individuals could confidently raise concerns, trusting in proper and fair handling.^38^ Moreover, dismantling hierarchical attitudes through open dialogue was suggested as something that could promote better teamwork and the sharing of experiences.^37^

In some organisations, breaking the prevailing culture of silence was highlighted as a key objective.^7,26,35^ Organisations should place greater emphasis on the safety of whistleblowers to overcome the culture of silence and encouraging open discussions can help overcome this culture.^35^ It is crucial that organisations respond appropriately to patient safety concerns and ensure that those who raise them are not blamed or penalised.^7^ Organisations should aim to establish an open and accountable culture that systematically promotes and values critical reflection and evaluation.^26^ At times, specific organisational units may be needed to foster an ethical culture in a particular work environment.^35^

A few suggestions for the development of whistleblowing practices emerged in this review. First, job rotation should be implemented, especially in leadership roles, to enhance the prevention and detection of misconduct.^35^ Second, misconduct should be handled collaboratively with colleagues and the work community. Addressing patient cases or organisational issues in groups can prevent staff isolation, empower staff, and reduce oppressive organisational cultures.^7^ Third, healthcare organisations could establish various tailored whistleblowing practices, depending on the situation.^35^ Organisations should ensure that appropriate channels are in place to investigate and respond to staff concerns related to care practices or safety issues.^7^ For instance, providing an anonymous reporting option could facilitate the raising of difficult issues, especially when interpersonal relationships and structural barriers are involved.^7,37^ Fourth, organisations should exhibit a genuine commitment to critically reviewing and, when necessary, adapting their whistleblowing practices and reporting systems.^26^

#### The need to reframe attitudes towards whistleblowing as both an opportunity and a tool for organisational improvement

The negative connotations attached to whistleblowing signal the need for a more positive framing of the concept. In organisations, leaders highlighted the importance of voicing safety and quality concerns; this emphasises that whistleblowing constitutes a vital instrument for risk management and is integral to organisational success.^35,38^ Viewed from this perspective, whistleblowing becomes crucial for improving efficiency, mitigating risks, fostering an ethical organisational culture, improving patient safety, and strengthening the relationship between patients and the organisation.^35^

Whistleblowing should be understood as a broad and comprehensive phenomenon.^26,30,35,36^ Structurally, it should be seen not only as a tool for risk prevention but also as a mechanism for quality improvement. Greater emphasis must be placed on the role and impact of whistleblowing in enhancing processes when designing healthcare plans and programmes.^35^ In considering regulatory boundaries and operational practices, it is essential to account for the multifaceted context of activities, rather than viewing whistleblowing as a binary choice between silence and action.^30^ Organisations should embed whistleblowing in accountability structures to enable risk assessment, encourage reporting and support barrier-free disclosures. Process leaders should be approachable and reliable and they should handle cases professionally.^26^ A unified organisational approach to systematically monitor deficiencies, identify trends, and implement corrective actions is essential. Without such an approach, healthcare systems cannot effectively learn from incidents.^36^

#### Summary of the structures and practices of whistleblowing in healthcare organisations

**Figure 2.**
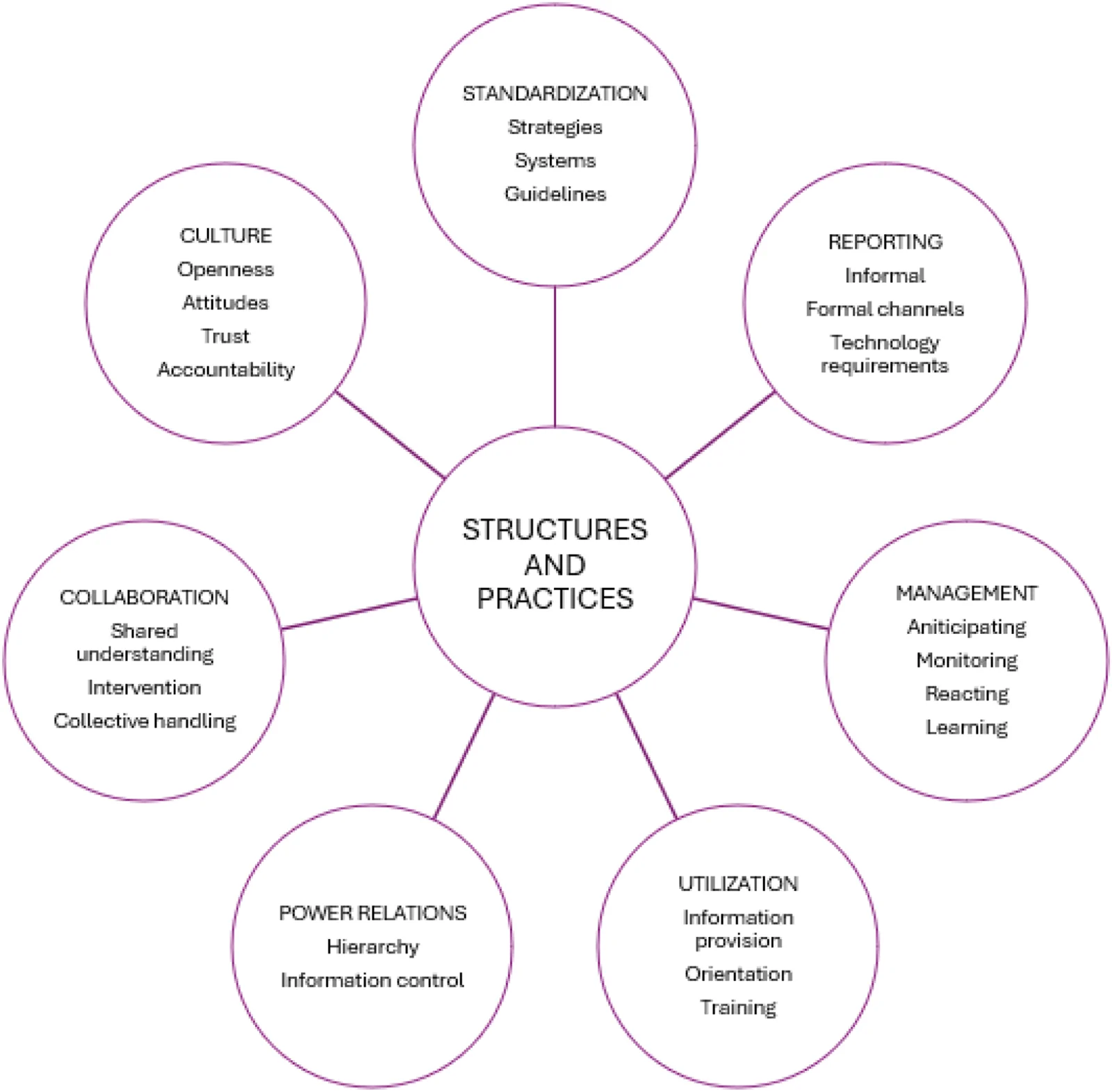
Structures and practices of whistleblowing in healthcare organizations.

The findings highlight a need for standardised whistleblowing strategies, systems, and guidelines to support systematic internal whistleblowing. Both informal opportunities for discussion and formal reporting channels, which should be supported by appropriate technological solutions, are essential for enabling reporting. From a managerial perspective, attention should be given to monitoring wrongdoing, whistleblowing, responding appropriately to reported concerns and learning from successfully resolved cases to anticipate and prevent future wrongdoing. Furthermore, effective utilisation of whistleblowing structures and practices requires comprehensive information provision, as well as adequate orientation and training for all healthcare workers. Power relations in organisations should be addressed by lowering hierarchical barriers and reducing excessive control over information. Whistleblowing also relies on collaboration, including shared understanding, collective interventions, and joint handling of whistleblowing cases. Finally, organisational culture should promote openness, positive attitudes towards whistleblowing, and trust and accountability in management (Figure 2).

## Discussion

In this review, we synthesised available knowledge on whistleblowing in healthcare from the perspective of organisations, which has been less studied. The previous reviews have mainly focused on individual whistleblowers, overlooking the structures and practices of whistleblowing. Although some scholars^12,39^ have described organisational responses and factors influencing whistleblowing, to the best of our knowledge, this is the first review focused on structures and practices of whistleblowing in the healthcare context. The knowledge provided here is useful for advancing internal whistleblowing and developing whistleblowing channels and processes in healthcare organisations.

Methodologically, conducting an integrative literature review enabled the combination of evidence obtained using different study designs^20^ to identify a broad range of structures and practices of whistleblowing in healthcare. In the reviewed articles, the prevalent study design was qualitative^7,33^; no quasi-experimental or randomised controlled trials were found. This highlights the need for more rigorous research to provide causal evidence and to evaluate the effectiveness of the structures and practices of whistleblowing, including the efficacy of whistleblowing technology and internal whistleblowing processes.

The findings show that structures and practices of whistleblowing in healthcare remain fragmented and ineffective,^35^and they are often shaped by organisational cultures and attitudes that are indifferent, hostile, or selectively responsive to concerns.^28^ Hostility towards whistleblowing has been consistently reported in the literature.^40,41^ These findings suggest that there is a need to develop interventions, such as ethics training, to foster more positive attitudes towards whistleblowing. Results also highlight systemic shortcomings in formal reporting structures, particularly from the user perspective,^38^ which have also been found in previous literature.^42^ Thus, there is a need to explore best practices pertaining to whistleblowing channels and technology solutions from the user perspective. Another systemic issue that prevents whistleblowing is the imbalance of power relations,^28,36,37^ which plays a central role in suppressing whistleblowing as democratic act and as a form of speaking truth to power.^43^ This problem is particularly evident in healthcare organisations, which have traditionally been hierarchical in structure.^44^ In previous research, such structural, cultural, and power-related barriers have been associated with prolonged organisational silence^13^ and ongoing ethical strain^19^ for healthcare professionals, emphasising the broader implications of ineffective whistleblowing practices.^12,19,40^

Despite legislative developments in various European countries following whistleblowing EU directive,^8^ findings of this review indicate that organisations may lack standardised and consistently implemented internal whistleblowing mechanisms, including options for anonymous reporting. This evidence is supported by a report^45^ showing that in most EU countries, whistleblower protection legislation does not fully comply with the relevant EU directive, nor does it meet the best practices of whistleblowing. Moreover, having whistleblowing legislation and channels in place is not sufficient on its own; clear protocols, procedures, and processes for responding to and resolving whistleblowing cases must also be developed. Without coherent organisational implementation, such arrangements risk remaining largely symbolic rather than effective in practice, thereby limiting their intended outcomes.^12,13^ In this regard, there is an opportunity to foster a learning culture centred on whistleblowing in healthcare organisations.^27^

The review findings show the influence of organisational culture and leadership on whistleblowing. If the prevailing organisational culture and leadership are tolerant of wrongdoing, there is the risk of a ‘slippery slope’ – a gradual erosion of ethical standards where deviant behaviours become normalised.^46^ This normalisation is especially influential for newcomers, who may initially question unethical practices but eventually come to accept them as standard because they are widely tolerated in the workplace.^30^ This cultural acceptance can undermine whistleblowing efforts and reinforce silence. Therefore, it is essential to nurture an organisational culture of integrity and accountability that demonstrates zero tolerance for wrongdoings.^47^ Whistleblowing should not be viewed only as an individual responsibility; rather, shared responsibility should also be promoted in healthcare organisations.^17^ Weaknesses in organisational culture and leadership with regard to whistleblowing hinder the ethical accountability of those in charge and compromise the quality of care, as well as the safety and well-being of patients and employees.^47^ The strengthening of structures and practices of whistleblowing requires procedural reforms, a cultural shift toward transparency, and openness.^30,32^ This includes fostering an environment where speaking up and discussions on ethical issues are supported.

Findings highlight a need for improvement of structures and practices of whistleblowing in healthcare organisations. This need was identified from stakeholder perspectives at all organisational levels – from individual healthcare professionals to working teams and organisational structures, as well as the broader societal context. Current whistleblowing systems remain incomplete and evolving.^26,30,35,36^ To ensure sustainability in organisations, whistleblowing must be normalised as part of everyday organisational life, and it must be supported by leadership that actively promotes ethical conduct and transparency. Training and induction processes play a critical role in equipping staff with the knowledge and confidence needed for whistleblowing. Reporting systems also require refinement to ensure accessibility, usability, and trustworthiness.^42^ Breaking the culture of silence is essential, and efforts should focus on creating supportive environments where speaking up is encouraged and protected.^48^ Moreover, the findings underscore the importance of reframing whistleblowing as both an opportunity and a strategic tool for organisational improvement.^35,38^ This involves shifting attitudes through positive framing, recognising whistleblowing as a broad and integrated phenomenon, and adopting a unified and systematic approach that embeds it into the core values and operational strategies of healthcare organisations.

### Limitations and strengths

This review has both limitations and strengths. Regarding the former, a single researcher performed the literature search. Another limitation is that only English-language articles were included, limiting linguistic diversity. The final limitation is that most of the studies were conducted in one country; however, several continents were represented. Thus, the review provided knowledge on the structures and practices of whistleblowing in healthcare systems across different cultures, which can be considered a strength. Another strength is the generally high quality of the included studies. The fact that the literature search strategy was developed in collaboration with a library informatics expert is also a strength of this review.

## Conclusions

This review provides an overview of whistleblowing in healthcare from an organisational perspective, a topic that has received limited attention in the previous research. The review reveals the fragmented and inconsistent nature of current structures and practices of whistleblowing in healthcare. It also highlights how organisational culture, leadership, and power dynamics shape the effectiveness of these structures and practices. The findings offer valuable development suggestions for healthcare organisations, particularly in regard to strengthening internal reporting mechanisms, fostering ethical leadership, and integrating whistleblowing into everyday practices through training and cultural reform. These implications are relevant to both general concern-raising systems and whistleblowing systems. From a policy perspective, the results can inform national and EU-level efforts to align legislation with best practices, especially in countries where whistleblower protection laws remain underdeveloped or poorly implemented. Whistleblowing must be recognised as a leadership issue and reframed as both an opportunity and a strategic tool for organisational learning, accountability, and improvement. Doing so will support ethically sustainable healthcare. Finally, concerning future research, this review identifies the need to obtain more robust evidence, particularly through longitudinal and intervention studies, in order to evaluate the effectiveness of whistleblowing structures, technologies, and organisational responses in healthcare settings.

## Supplemental material

Supplemental material - Structures and practices of whistleblowing in healthcare: An integrative review Supplemental material for Structures and practices of whistleblowing in healthcare: An integrative review by Outi Järvinen, Riitta Suhonen, and Johanna Wiisak in Nursing Ethics

## Data Availability

Data sharing is not applicable, as this integrative literature review is based solely on previously published data.*
