# Supplementary material for: Structures and practices of whistleblowing in healthcare: An integrative review
Source: Nurs Ethics. 2026 May 20;33(6):1871–91. doi: 10.1177/09697330261449295 (PMC13522412; doi:10.1177/09697330261449295)
Supplement: Supplementary file 1 [file sj-pdf-1-nej-10.1177_09697330261449295.pdf]

Supplementary file 1. Databases and search strings and hits (n =)

| **Database** | **Search strings** | **Hits (n =)** |
|---|---|---|
| PubMed | PubMed: ("Whistleblowing"[Mesh] OR whistleblow* OR ”whistle-blow*” OR "whistle blow*") AND ("Organizational Policy"[Mesh] OR "organizational policies" OR ”organisational policies” OR "organizational policy" OR ” organisational policy” OR ”Practice pattern*” OR "organizational practic*" OR ”organisational practic*” OR "organizational structur*" OR ”organisational structur*” OR "organizational channel*" OR ”organisational channel*” OR "organizational consequenc*" OR ”organisational consequenc*” OR "organizational program*" OR ”organisational program*” OR "organizational intervention*" OR ”organisational intervention*” OR "organizational process*" OR ”organisational process*” OR "organizational system*" OR ”organisational system*” OR "organizational report*" OR ”organisational report*” OR "organizational cultur*" OR ”organisational cultur*”) AND (healthcare* OR ”health-care*” OR ”health care*”) | 76 |
| CINAHL | ((MH "Whistle Blowing") OR whistleblow* OR ”whistle-blow*” OR "whistle blow*") AND ((MH "Organizational Policies+") OR (MH "Practice Patterns") OR "organizational policies" OR ”organisational policies” OR "organizational policy" OR ”organisational policy” OR "organizational practic*" OR ”organisational practic*” OR "organizational structur*" OR ”organisational stuctur*” OR "organizational channel*" OR ”organisational channel*” OR "organizational consequenc*" OR ”organisational concequenc*” OR "organizational program*" OR ”organisational program*” OR "organizational intervention*" OR ”organisational intervention*” OR "organizational process*" OR ”organisational process*” OR "organizational system*" OR ”organisational system*” OR "organizational report*" OR ”organisational report*” OR "organizational cultur*" OR ”organisational cultur*”) AND (healthcare* OR ”health-care*” OR ”health care*”) | 89 |
| SocINDEX | ((DE "WHISTLEBLOWING") OR whistleblow* OR ”whistle-blow*” OR "whistle blow*") AND (OR "organizational policies" OR ”organisational policies” OR "organizational policy" OR ”organisational policy” OR "organizational practic*" OR ”organisational practic*” OR "organizational structur*" OR ”organisational structur*” OR "organizational channel*" OR ”organisational channel*” OR "organizational consequenc*" OR ”organisational concequenc*” OR "organizational program*" OR ”organisational program*” OR "organizational intervention*" OR ”organisational intervention*” OR "organizational process*" OR ”organisational process*” OR "organizational system*" OR ”organisational system*” OR "organizational report*" OR ”organisational report*” OR "organizational cultur*" OR ”organisational cultur*”) AND ((DE "HEALTH facilities") OR healthcare* OR ”health care*” OR ”health-care*”) | 3 |
| Web of Science | (whistleblow* OR ”whistle-blow*” OR "whistle blow*") (Topic) AND ("organizational policies" OR ”organisational policies” OR "organizational policy" OR ”organisational policy” OR ”practice pattern*” OR "organizational structur*" OR ”organisational structur*” OR "organizational channel*" OR ”organisational channel*” OR "organizational consequenc*" OR ”organisational concequenc*” OR "organizational program*" OR ”organisational program*” OR "organizational intervention*" OR ”organisational intervention*” OR "organizational process*" OR ”organisational process*” OR "organizational system*" OR ”organisational system*” OR "organizational report*" OR ”organisational report*” OR "organizational cultur*" OR ”organisational cultur*”) (Topic) AND (healthcare* OR "health-care*" OR ”health care*”) (Topic) | 17 |
| Scopus | Scopus: (whistleblow* OR ”whistle-blow*” OR "whistle blow*") AND ("organizational policies" OR ”organisational policies” OR "organizational policy" OR ”organisational policy” OR ”practice pattern*” OR "organizational structur*" OR ”organisational structur*” OR "organizational channel*" OR ”organisational channel*” OR "organizational consequenc*" OR ”organisational concequenc*” OR "organizational program*" OR ”organisational program*” OR "organizational intervention*" OR ”organisational intervention*” OR "organizational process*” OR "organisational process*” OR "organizational system*" OR ”organisational system*” OR "organizational report*” OR "organisational report*” OR "organizational cultur*" OR ”organisational cultur*”) AND (healthcare* OR ”health-care*” OR ”health care*”) | 94 |
| **Hits total** | | 279 |
